# Physiological and Behavioral Changes in Honey Bees (*Apis mellifera*) Induced by *Nosema ceranae* Infection

**DOI:** 10.1371/journal.pone.0058165

**Published:** 2013-03-06

**Authors:** Mike Goblirsch, Zachary Y. Huang, Marla Spivak

**Affiliations:** 1 Department of Entomology, University of Minnesota, St. Paul, Minnesota, United States of America; 2 Department of Entomology, Michigan State University, East Lansing, Michigan, United States of America; Arizona State University, United States of America

## Abstract

Persistent exposure to mite pests, poor nutrition, pesticides, and pathogens threaten honey bee survival. In healthy colonies, the interaction of the yolk precursor protein, vitellogenin (Vg), and endocrine factor, juvenile hormone (JH), functions as a pacemaker driving the sequence of behaviors that workers perform throughout their lives. Young bees perform nursing duties within the hive and have high Vg and low JH; as older bees transition to foraging, this trend reverses. Pathogens and parasites can alter this regulatory network. For example, infection with the microsporidian, *Nosema apis*, has been shown to advance behavioral maturation in workers. We investigated the effects of infection with a recent honey bee pathogen on physiological factors underlying the division of labor in workers. Bees infected with *N. ceranae* were nearly twice as likely to engage in precocious foraging and lived 9 days less, on average, compared to controls. We also show that Vg transcript was low, while JH titer spiked, in infected nurse-aged bees in cages. This pattern of expression is atypical and the reverse of what would be expected for healthy, non-infected bees. Disruption of the basic underpinnings of temporal polyethism due to infection may be a contributing factor to recent high colony mortality, as workers may lose flexibility in their response to colony demands.

## Introduction

The economic and ecological importance of honey bees (*Apis mellifera*) as pollinators of many cultivated and native plants make them an important system for studying the effects of illness at both the individual and colony or social levels. In the U.S. and worldwide, it has become increasingly difficult to keep colonies alive as bees are challenged with numerous factors that threaten their survival. Mite pests, pathogens, pesticides, and nutritional deficiencies create a combination of circumstances that can interact negatively to jeopardize colony health [1], [2]. For the U.S. specifically, the outcome of this health crisis has been losses of nearly one-third of colonies annually since 2006 [3]. This recurring level of death may be unsustainable for the beekeeping industry, and could debase the value of crops and other products requiring pollination [4].

Many pathogens and parasites are common and widespread in non-symptomatic, or apparently healthy, colonies [5]. This burden emphasizes the buffering capacity of honey bee societies, but makes it difficult to state with confidence the impact a specific factor has on colony health. Fortunately, a wealth of research is available describing physiological factors that influence the age-based division of labor of workers within healthy colonies [reviewed by: 6,7]. We relied on this research background to explore mechanisms that could interfere with worker behavior following exposure to a single specific pathogen, *Nosema ceranae*, considered to be a factor in colony decline [1].

The honey bee worker caste displays an age-based division of labor. In northern temperate climates, workers that emerge as adults during the summer live 6 wks on average. Upon emergence, workers less than 3 wks of age remain in the hive as nurses, feeding the larvae and queen, or performing nest maintenance. At about 3 wks, workers transition from nursing duties to foraging for nectar and pollen outside the nest. Once workers transition to foraging, they typically live an additional 2−3 wks, regardless of the age at which the shift occurs [8]. To maintain colony cohesion, workers demonstrate a level of flexibility by advancing, delaying, or reverting behavioral development in response to the needs of the colony such as a loss of foragers from predation or confinement of foragers during inclement weather [9]–[11].

The yolk precursor protein, vitellogenin (Vg), and endocrine factor, juvenile hormone (JH), are thought to be physiological regulators underlying behavioral development in honey bee workers [12]–[14]. Vitellogenin has multiple roles in honey bee health; it is essential for egg development, has immune response and antioxidant properties, and serves as a nutrient reserve and lipid carrier [15]. Nurse bees have high Vg in fat body tissues and low JH in hemolymph, whereas at the onset of foraging, older bees have low Vg and high JH [11], [16]. The regulatory interaction between Vg and JH has been demonstrated experimentally in nurse-aged bees using RNA interference, where knockdown of Vg expression resulted in increased JH titer, rapid foraging onset, and a shortened lifespan [17]. Moreover, topical application of JH has been shown to induce precocious foraging [18].

*Nosema ceranae* is a spore-forming fungus that infects and reproduces inside epithelial cells of the midgut. During the course of infection, millions of spores are produced and released into the environment when a bee defecates. These spores are a source of infection for other bees. For a colony containing thousands of workers, infection and untimely death of one bee may be an insignificant loss. However, as infection spreads to a large number of bees a cascade effect could ensue to challenge colony survival: as the infected population dies, the queen may be unable to lay enough eggs to replace the loss, and remaining workers may lose flexibility in their behavioral development to respond to task demand.

Studies detailing the pathological effects of *N. ceranae* on individual workers have focused mainly on consequences to mortality [19], [20]. Some studies have examined the effects of infection on expression of immune-related peptides [21], [22] or looked at changes in nutritional or energetic states [23], [24]. However, our understanding of underlying physiological effects contributing to the disease process is incomplete. Moreover, behavioral studies linking changes seen in the physiology of infected bees are lacking and would help ascertain effects to host and/or colony fitness. We observed bees infected with *N. ceranae* in field colonies to establish the age of foraging onset. We then measured physiological responses, specifically Vg and JH, of infected bees in cages and field colonies. We used qRT-PCR and a radioimmunoassay to measure relative Vg transcript in tissues and JH titer in hemolymph, respectively. Our objective was to determine whether infection with *N. ceranae* disrupts fundamental physiological processes underlying ancestral reproductive traits that honey bees co-opted as a mechanism to control social behavior [11], [13].

## Materials and Methods

### Ethics Statement

No specific permits were required for the described field studies. Observations were conducted at the University of Minnesota apiary; therefore, no specific permissions were required for this location. The apiary is the property of the University of Minnesota and not privately-owned or protected in any way. Field studies involved observing the European honey bee (*Apis mellifera*), which is neither an endangered or protected species.

### Spore Purification

*Nosema ceranae* spores were procured for infection studies by Percoll purification. Briefly, bees were collected and anesthetized on ice from a colony known to contain individuals infected with *Nosema* sp. as determined by microscopy. The alimentary tract was removed from a sufficient number of bees to ensure a large source of inoculum. The alimentary tracts were pooled in a sterile 50 mL conical tube containing 15 mL of sterile water and homogenized using a Polytron tissue homogenizer (Brinkmann Instruments, Westbury, NY). The homogenate was passed through a gauze filter to remove debris and then brought to a volume of 40 mL with sterile water. The suspension was centrifuged at 1500 g for 10 min at 4°C. The supernatant was discarded and the pellet was resuspended by vortexing in 40 mL of sterile water, followed by centrifugation at 1500 g for 10 min at 4°C. This step was repeated 2 times. The supernatant was discarded and the pellet was resuspended by vortexing in 20 mL of 100% Percoll (Sigma-Aldrich, St. Louis, MO) adjusted to a pH of 7.0 with 1 N HCl. The suspension was centrifuged at 2500 g for 30 min at 4°C. The Percoll column was carefully removed and discarded, and the pellet was washed once with 5 mL of sterile water. The final pellet was resuspended in sterile water and stored at 4°C until use. Confirmation to species was performed by conventional PCR using previously described primers [25].

### Inoculation of Bees

Frames of capped brood were removed from colonies with no known, apparent symptoms of disease, and incubated overnight at 32°C and 70% relative humidity (RH). The following day, bees that had emerged were collected from frames within 24 h after eclosion. Each bee that was to be inoculated was restrained between the thumb and forefinger by laboratory staff and orally administered 10^4^ *N. ceranae* spores in 5 µL of sucrose solution (50% w:v) or an equal volume of sucrose solution without spores using micropipettes. Bees were held individually in 20 mL vials after inoculation for ≥20 min to ensure the inoculum was ingested. Bees that did not consume the inoculum were discarded.

### Mortality Assessment

Bees were inoculated within 24 h after eclosion as above and put in 11×11×11 cm cages according to experimental group along with 30 paint-marked un-inoculated bees to provide social interaction and serve as controls that received minimal handling. Cages were placed in a darkened environmental chamber maintained at 28°C and 70% RH. Bees were given *ad libitum* access to 50% sucrose solution and water supplied in gravity feeders, and 5 g MegaBee protein patty (Dadant & Sons, Inc., Hamilton, IL) placed in a weigh boat. Cages were rotated daily to minimize position effects, and sucrose, water, and protein patty were replaced weekly. Mortality in each cage was recorded and dead bees were removed daily. Three replicate cages of 30 bees, except cage 3 from the infected group that contained 33 bees, were tested for both infected and control conditions. Mortality results are presented as the daily mean percent survival.

### Behavioral Observations

To determine whether infection leads to precocious foraging, bees were inoculated within 24 h after eclosion with 10^4^ *N. ceranae* spores or sucrose solution as above, except trial 2 where *Nosema*-infected bees received 10^5^ spores per bee. Bees were paint-marked to identify treatment and introduced into field colonies containing an egg-laying queen and 5 frames equalized for brood, food, and adults. One hundred bees per treatment were introduced into each of 8 field colonies for trial 1 and 2 and each of 10 field colonies for trial 3. Typically, bees transition from in-hive tasks to foraging behavior at 3 wks of age; therefore, foraging behavior was observed daily from 7 until 21 days of age, which was considered as the precocious window for this behavior. Foraging was observed during a 30-min observation session by blocking colony entrances with 8 mesh hardware cloth and recording all paint-marked bees that returned. Only paint-marked bees with pollen loads in their corbiculae were considered as foragers. Marked foragers were removed upon appearance; however, other marked bees not showing evidence of foraging were not collected and may have been counted on subsequent days. The foraging assay was conducted over 3 trials, twice in 2010 (July and August) and once in 2011 (July).

### Sample Collection for Physiological Measures

Bees received either 10^4^ *N. ceranae* spores or sucrose solution within 24 h after eclosion as above and were placed in cages containing paint-marked bees that were not inoculated to provide social interaction. Un-inoculated bees were members of the same cohort as inoculated bees, but received minimal handling. Eight replicate cages containing 120 inoculated and 80 un-inoculated bees per cage were established per treatment. Dead bees were removed regularly and cages were maintained as above.

At 4, 8, 12, and 16 days of age, 8–10 bees were randomly selected and removed from each cage. Selected bees were grouped by cage into 15 mL sterile conical tubes on ice to induce anesthetization. Anesthetized bees were immobilized and ≥1 µL of hemolymph was obtained from each bee by piercing the intersegmental membrane between the second and third abdominal tergites and drawing hemolymph up by capillary action using a Drummond Wiretrol (Drummond Scientific Company, Broomall, PA). Hemolymph was discharged into a 12×125 mm glass tube with Teflon-lined cap containing 500 µL of chilled acetonitrile and stored at −20°C until JH analysis. The sting apparatus and as much of the attached alimentary tract as possible was removed by grasping it with forceps and pulling away from the terminal abdominal segments. The sting apparatus and attached alimentary tissues were placed in 1 mL of sterile water in a microcentrifuge tube and kept at 4°C until inspection for spores by microscopy. Next, the head was severed and discarded, and the remaining carcass was put into a microcentrifuge tube and flash frozen in liquid nitrogen and stored at −80°C until Vg analysis.

To compare levels of Vg and JH in infected versus control bees under natural conditions, an additional 80 bees per treatment were introduced into each of 10 field colonies containing a queen and equal amounts of brood, food, and adults. Field colonies were the same as those used in trial 3 for foraging observations. Bees were inoculated with either 10^4^ *N. ceranae* spores or sucrose within 24 h after eclosion as above and given paint marks to allow identification by treatment. At 7 and 15 days of age, 4 bees per treatment were collected on ice from each colony. Bees were processed for infection status, hemolymph collection, and tissue processing as above. Only those bees from the *Nosema*-infected group that showed presence of spores in gut homogenates by microscopy were used for Vg and JH analysis (Table 1). Absence of spores in gut homogenates of sucrose controls was also confirmed by microscopy before further analysis for Vg and JH.

**Table 1:** Mean number of spores per bee (×10^6^) at different ages after eclosion for bees used for Vg and JH analysis from cage and field studies.

Source of infected bees	Mean spores per bee ± SE (×10^6^)			
Cage studies (days of age from eclosion)	4	8	12	16
	0.1±0.0	6.1±0.8	29.7±2.3	27.4±4.3
Field studies (days of age from eclosion)	7	15		
	0.5±0.1	17.4±3.4		

### Radioimmunoassay to Quantify JH

A chiral-specific radioimmunoassay was used to quantify JH titer in hemolymph collected from individual bees [16]. One mL of hexane and 0.5 mL of 0.9% NaCl were added to samples and centrifuged at 2000 g for 10 min. The JH-containing hexane phase was transferred to a new tube. This process was repeated to increase extraction efficiency. Hexane was evaporated using a Savant drying system (Thermo Fischer Scientific, Inc., Pittsburgh, PA). Dried samples were chilled on ice and washed with 100 µL of methanol by vortexing. A 10 µL aliquot was transferred to a new tube and the methanol was evaporated. Next, 200 µL of premixed JH antiserum (1∶28,000) and 10,000 DPM [10-3H(N)]-JH (Perkin Elmer, Inc., Waltham, MA) were added to the tubes. The tubes were incubated for 2 h, when radiolabeled JH and JH in the hemolymph competed for binding to the antibody. The reaction was slowed by chilling the tubes in ice water for 10 min. To absorb unbound JH, samples were incubated in 500 µL of dextran-coated charcoal for 2.5 min. The tubes were centrifuged at 2000 g for 3 min to pellet the charcoal. The supernatant containing bound JH was decanted into 20 mL scintillation vials and 5 mL of Ultima Gold scintillation cocktail (Perkin Elmer, Inc., Waltham, MA) was added. Radioactivity was quantified and values were evaluated against standards fitted by non-linear regression. Samples were run in duplicate. All glassware was baked at 500°C for 3.5 h prior to use to minimize JH adsorption, and all reagents were HPLC grade. The JH antiserum was provided by David Borst (Central Florida University).

### qRT-PCR for Relative Vg Gene Expression

Total RNA was extracted using an RNAqueous kit (Ambion, Austin, TX) for cage studies or TRIzol method for field studies. DNA contamination was degraded with 10 U DNAse I (Ambion, Austin, TX) at 37°C for 1 h followed by 75°C for 10 min. cDNA was synthesized by incubating 8 µL total RNA with mastermix containing 50 U Superscript II reverse transcriptase (Invitrogen, Carlsbad, CA), 20 U RNaseOUT ribonuclease inhibitor (Invitrogen, Carlsbad, CA), 2 µM dNTPs, 2 nM poly(dT)_18_, and 0.1 nM poly(dT)_12–18_ at 42°C for 50 min followed by 15 min at 70°C as described previously [26].

Transcript was assayed by qPCR. Two µL of cDNA served as template in a reaction containing 1 U Taq polymerase, 1× buffer, 1 mM dNTPs, 2 mM MgCl_2_, 0.2 µM of forward (5′-agttccgaccgacgacga-3′) and reverse (5′-ttccctcccacggagtcc-3′) primers (Genbank Entry: NP_001011578) specific for amplification of the honey bee Vg precursor gene, and 1× SYBR-Green (Applied Biosystems, Foster City, CA) for a total volume of 25 µL. Reactions were run using a thermal profile consisting of 95°C for 30 sec, followed by 40 cycles of a 4-step protocol consisting of 95°C for 20 sec, 60°C for 30 sec, 72°C for 1 min, and 78° for 20 sec. Fluorescence measurements were taken repeatedly during the 78°C step. A 3-step melt curve was performed to ensure proper amplification of the target gene. The reference gene, β*-*actin, was amplified with forward (5′-ttgtatgccaacactgtccttt-3′) and reverse (5′-tggcgcgatgatcttaattt-3′) primers (Genbank Entry: NC_007076) to normalize data according to RNA amount in each sample. All primers were published previously and validated experimentally [26], [27].

### Statistical Analyses

Data were analyzed using R version 2.13.0 (2011-04-13) statistical software with car package. Mortality data were analyzed by Cox proportional-hazards regression model with splines package. Differences in the number of returning marked pollen foragers were analyzed by Chi-squared test. Differences in Vg transcript and JH titer were analyzed by mixed model ANOVA with nlme package. Infection status and age of bees were modeled as fixed effects and cage or colony of origin were modeled as random effects. JH data were natural logarithm transformed to meet assumptions of a normal distribution for residuals and to stabilize variances. All statistical tests used α = 0.05 to establish significance. Data are reported as means ± SE.

## Results

### Infected Bees have a Shorter Lifespan Compared to Controls

**Figure 1:**
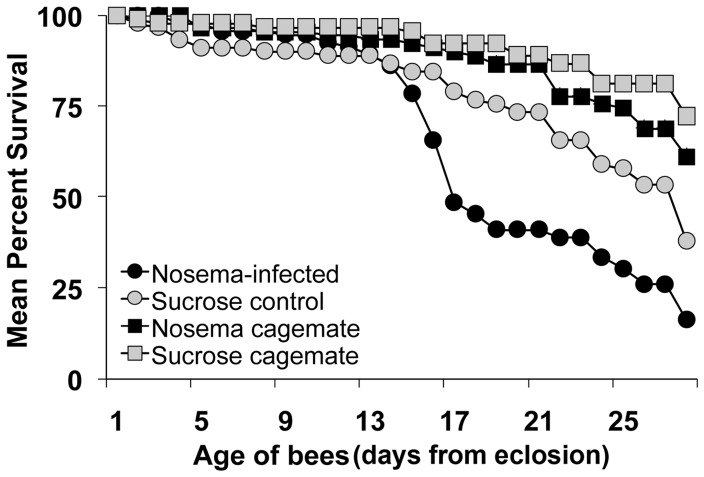
*Nosema ceranae* shortens honey bee lifespan. Mean survival (%) of caged bees fed 10^4^ *N. ceranae* spores (*Nosema-*infected) or sucrose solution (Sucrose control) at day 0 (i.e., within 24 h after adult emergence). Age-matched cagemates that were not inoculated received minimal handling and were placed into infected (*Nosema* cagemate) or control (Sucrose cagemate) cages to simulate social interaction. n = 30 bees per treatment/cage (except cage 3 from the infected group that had 33 bees), 3 replicate cages per treatment. Infection had a significant negative effect on lifespan (*z* = 3.97, n = 363, *p*<0.0001).

*Nosema ceranae* had a negative effect on lifespan (*z* = 3.97, n = 363, *p*<0.0001; Figure 1). Infection increased the daily mortality by a factor of 1.99. Survivorship was above 75% and indistinguishable between infected and control bees from 1 through 14 days of age. However, the number of surviving infected bees dropped below 50% by 16 days of age, whereas controls did not reach this level until 25 days of age. Therefore, most control bees lived, on average, 9 days longer than infected bees.

To account for mortality associated with handling during inoculation, an additional 30 bees that received minimal handling were put into each cage (i.e., un-inoculated cagemates). Daily mortality was significantly greater for bees that received handling during inoculation compared to un-inoculated cagemates (*z* = 4.46, n = 363, *p*<0.0001). Handling increased the daily mortality by a factor of 2.93. Un-inoculated cagemates for both infected and control conditions had survivorship levels ≥60% throughout the experiment. There was no difference in the daily mortality rate between un-inoculated cagemates in *Nosema*-infected or control cages (*z* = 1.59, n = 363, *p* = 0.11).

### Infection with *N. ceranae* Results in Premature Foraging

**Figure 2:**
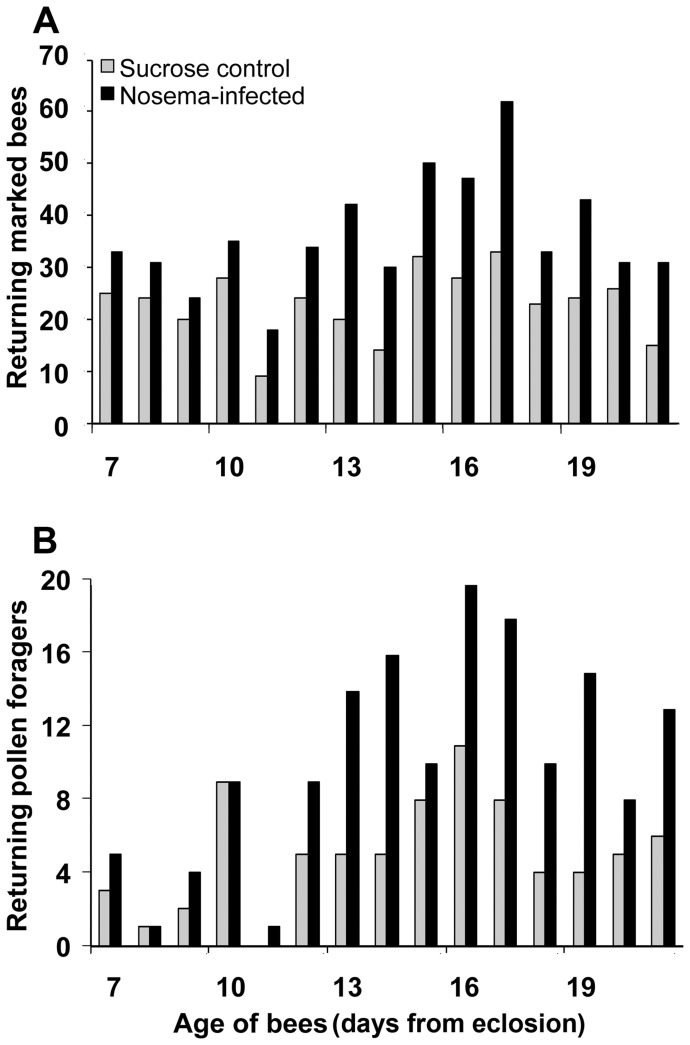
Honey bees infected with *N. ceranae* forage prematurely. A. Distribution of returning marked bees during a daily 30-min observation period from 7 through 21 days of age after eclosion. Data are summarized over 3 independent trials. **B.** Distribution of returning marked pollen foragers collected daily during a 30-minute observation period from 7 through 21 days of age after eclosion. Data are summarized over 3 independent trials. Significantly more marked pollen foragers were collected from the *Nosema*-infected group compared to controls for the 3 trials combined (χ^2^ = 26.38, *p*<0.0001).

A greater number of infected marked bees compared to non-infected marked bees were recorded at entrances to field colonies starting at 7 through 16 days of age (Figure 2A). There were ≥30 marked bees recorded on 13 of 15 days for the infected group compared to only 2 of 15 days for controls. In all, there were 206, 79, and 261 marked bees (total = 546) recorded for the infected group compared to 130, 64, and 151 marked bees (total = 345) for sucrose controls for trials 1, 2, and 3, respectively (Table 2).

**Table 2:** The number of returning marked bees, and of those, the number of returning pollen foragers observed at entrances to field colonies during daily 30-min observations for 3 independent trials.

	Marked returning bees		Marked pollen foragers	
Trial	Control	Infected	Control	Infected
1. July, 2010	130	206	38^a^	73^b^
2. August, 2010	64	79	15^a^	29^b^
3. July, 2011	151	261	23^a^	51^b^
Total	345	546	76^a^	153^b^

Bees were introduced into field colonies after receiving either *N. ceranae* spores (Infected) or sucrose solution (Control) within 24 h after eclosion. Marked bees were counted once during the daily observation period. Marked foragers were collected without replacement. Counts between rows within category followed by different letters are significant for the marked pollen forager data (Chi-squared test, *p*<0.05).

It is unknown whether all returning marked bees were engaged in foraging; therefore, only bees carrying loads of pollen were recorded as foragers for the following analysis. The distribution shows that there were ≥10 foragers recorded on 8 of 15 days for the infected group compared to only 1 of 15 days for controls. Foragers from the infected group outnumbered controls at nearly 2 to 1 for each trial and the 3 trials combined. In all, there were 73, 29, and 51 foragers (total = 153) for the infected group compared to 38, 15, and 23 foragers (total = 76) for sucrose controls for trials 1, 2, and 3, respectively. Chi-squared analysis showed significantly more foragers were collected from the infected group for trial 1 (χ^2^ = 11.19, *p* = 0.0008), trial 2 (χ^2^ = 3.95, *p* = 0.047), trial 3 (χ^2^ = 10.23, *p* = 0.0014), and the 3 trials combined (χ^2^ = 26.38, *p*<0.0001) compared to controls. For trial 2, all colonies were inspected on days 4, 12, and 21 after the introduction of paint-marked bees and it was noted that there were relatively equal numbers of bees from both treatment groups present. Therefore, we assume that uninfected control bees did not die at a faster rate than *Nosema-*infected bees during the field trials, which is in agreement with our mortality assessment.

### Infection has a Negative Effect on Vg Transcript Over Time for Bees in Cages, but not Field Conditions

**Figure 3:**
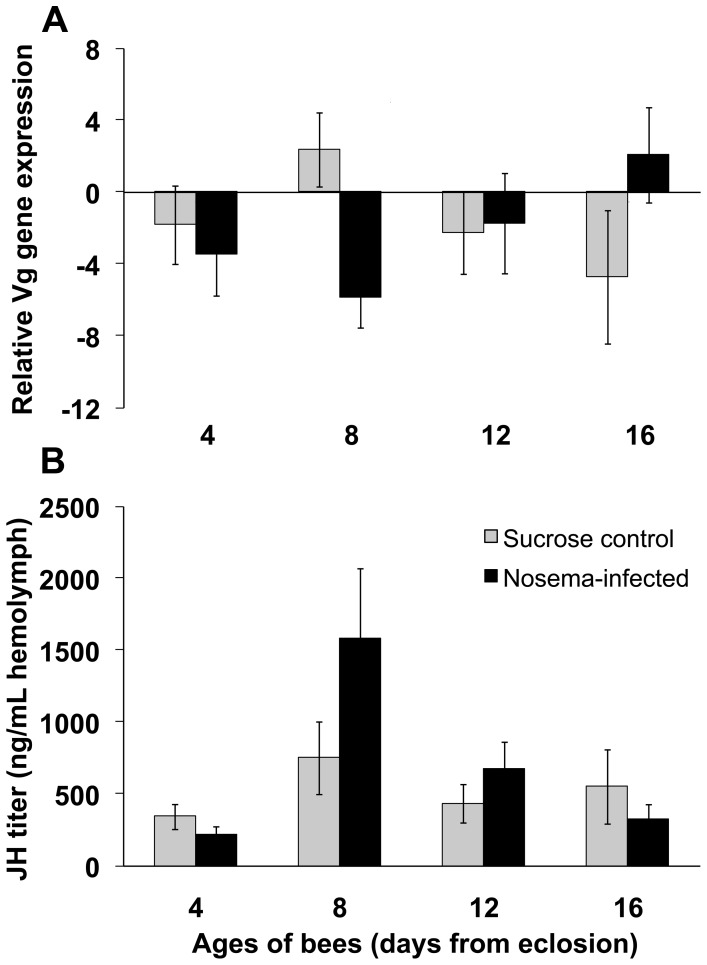
*Nosema ceranae* disrupts honey bee behavioral physiology. A. Vitellogenin transcript normalized to the reference gene, β-actin, (mean ± SE) for caged bees fed *N.* ceranae spores (*Nosema-*infected) or sucrose solution (Sucrose control) at day 0 (i.e., within 24 h after adult emergence). n≥17 per treatment per day. There was a significant interaction between infection status and age of bees on relative Vg transcript (*F*_3,191_ = 3.04; *p* = 0.030). **B.** JH titer (mean ± SE) for caged bees fed *N.* ceranae spores (*Nosema-*infected) or sucrose solution (Sucrose control) at day 0 (i.e., within 24 h after adult emergence). n≥20 per treatment per day. The interaction effect of infection status on JH titer over time was not significant (*F*_3,212_ = 2.00, *p* = 0.11).

There was a significant interaction between infection status and age of bees on relative Vg transcript in caged bees (*F*_3,191_ = 3.04; *p* = 0.030; Figure 3A). Control bees exhibited a normal Vg profile for adult worker ontogeny [11]: relative levels peaked at 8 days of age and then decreased linearly through 16 days of age. *Nosema*-infected bees showed the opposite trend: relative levels were low at 4 and 8 days of age with a 1.9-fold and 3.5-fold decrease in Vg, respectively, compared to control bees. After day 8, Vg levels increased linearly for infected bees. At 16 days of age, infected bees had a 3.3-fold increase in relative Vg compared to age-matched controls. The main effects of infection status or age of bees on relative Vg transcript were not significant (*p*>0.05).

**Figure 4:**
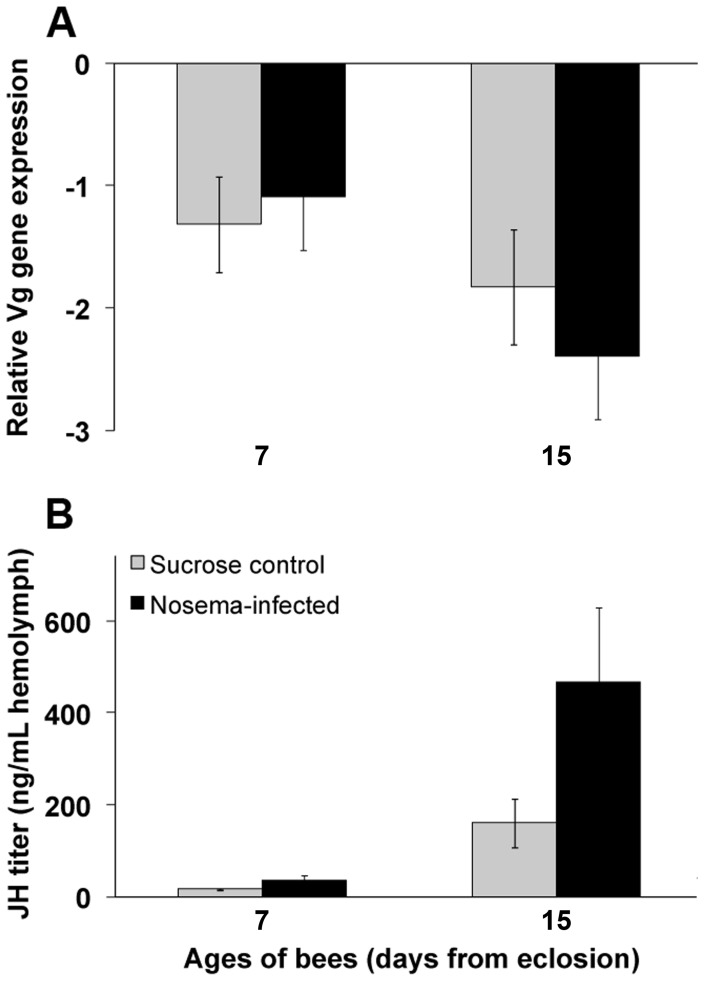
Honey bees infected with *N. ceranae* have elevated JH titer under field conditions. A. Vitellogenin transcript normalized to the reference gene, β-actin, (mean ± SE) for bees fed *N.* ceranae spores (*Nosema-*infected) or sucrose solution (Sucrose control) at day 0 (i.e., within 24 h after adult emergence) and placed into field colonies. n≥22 per treatment per day. There was no significant interaction effect of *N. ceranae* infection on relative Vg transcript over time (*F*_1,80_ = 0.80; *p* = 0.37). **B.** JH titer (mean ± SE) for bees fed *N. ceranae* spores (*Nosema-*infected) or sucrose solution (Sucrose control) at day 0 (i.e., within 24 h after adult emergence) and placed into field colonies. n≥31**per treatment per day. The effect of infection status on JH titer was marginally significant (*F*_1,119_ = 3.81, *p* = 0.053).

Levels of relative Vg for infected bees from field colonies were not different from controls (*F*_1,80_ = 0.22; *p* = 0.64; Figure 4A). However, there was an effect of age of bees on Vg transcript (*F*_1,80_ = 4.91; *p* = 0.030). As would be expected, levels of Vg decreased with the age of the worker. Transcript abundance was comparable between controls (−1.32 Vg units) and infected bees (−1.09 Vg units) at 7 days of age but decreased by 1.4-fold and 2.2-fold by 15 days of age, respectively. There was no interaction between infection status and age of bees on relative Vg transcript (*F*_1,80_ = 0.80; *p* = 0.37).

### Change in JH Titer in Hemolymph of Bees in Cages and Field Conditions

Infection did not show an effect on JH titer of bees in cages (*F*_1,14_ = 0.47, *p* = 0.51). However, infected bees had a 1.4-fold increase in JH titer, on average, compared to controls throughout the time course of the study (Figure 3B). The trend for bees from the infected group revealed a spike in JH titer in 8-day-old bees (1580.18 ng/mL), which was an increase of 2.1-fold compared to the average level for 8-day-old control bees (750.09 ng/mL). Juvenile hormone titer showed a linear decline from 8 days of age through 16 days of age for the infected group. In contrast, JH titer remained static throughout the time course for control bees. Bees older than 16 days of age were not collected; therefore, it is unknown whether JH titer for controls would have been elevated as predicted for workers ≥3 wks old [11]. There was a significant effect of age on JH titer (*F*_3,212_ = 8.22, *p*<0.0001). Eight-day-old bees in cages had at least a 2-fold increase in average JH titer compared to bees at the other time points. There was no interaction between infection status and age of bees in cages on JH titer (*F*_3,212_ = 2.00, *p* = 0.11).

Infected bees in field colonies had nearly a 3-fold increase in average JH titer compared to controls (Figure 4B); this difference was marginally significant (*F*_1,119_ = 3.81; *p* = 0.053). Juvenile hormone was low for both infected and control bees at 7 days of age, but infected bees had a 2.2-fold increase in JH titer in hemolymph (34.10 ng/mL) compared to age-matched controls (15.67 ng/mL). Juvenile hormone titer for infected bees rose sharply at 15 days of age and was 2.9-fold greater than age-matched controls. There was a significant difference in JH titer among bees of different ages in field colonies (*F*_1,119_ = 100.21, *p*<0.0001). As would be expected for aging workers, 15-day-old bees averaged a 12.7-fold increase in JH titer compared to 7-day-old bees. The interaction between infection status and age of bees on JH titer was not significant (*F*_1,119_ = 0.95; *p* = 0.33).

## Discussion

*Nosema ceranae* is a common and widespread pathogen of honey bees in the U.S. and worldwide [28], [29]. In this report, we demonstrate that *N. ceranae* can trigger premature foraging and shorten the lifespan of infected workers. We also show that Vg transcript was low, while JH titer spiked, in infected nurse-aged bees. This pattern is atypical and the reverse of what would be expected for healthy, non-infected bees. In honey bees, the transition from nursing inside the brood nest to foraging outside the hive is thought to be under regulatory control through the interaction of the yolk precursor protein, Vg, and endocrine factor, JH [9]. This interaction serves as an underlying physiological mechanism that is essential for allowing plasticity in behavioral responses by workers to dynamic colony and ecological environments [7]. In *Nosema*-infected bees, this regulatory framework is apparently disrupted.

In our experiment examining the effects of infection on lifespan, we found that controls lived, on average, 9 days longer than infected bees. During summer months, a typical worker bee lives 3−6 wks [30], with the final 2−3 wks devoted to foraging [8]. Assuming there is a similar mortality rate due to infection in field colonies, decreasing worker lifespan by 9 days, especially during the foraging phase, can be significant. Any nectar and/or pollen that would have been collected by the infected bee during these 9 days of life would be lost. Moreover, if the infected bee was part of the colony’s foraging force that performs scouting behavior for novel floral patches [31], the loss could be compounded as information detailing patch location would not be communicated.

Parasites can accelerate or delay host development as a strategy that exploits host resources and maximizes transmission [32]. Lengthening the immature development of insect hosts has been demonstrated with other *Nosema* spp. [33], [34]. In contrast, our findings suggest that *N. ceranae* accelerates honey bee behavioral development. We observed a greater number of infected bees outside the nest and engaged in foraging compared to controls. Evidence from other studies has shown anatomical or behavioral changes that are congruent with accelerated development resulting from infection with a similar pathogen, *N. apis*. Bees infected with *N. apis* were found to have reduced size and function of hypopharyngeal glands, which secrete a protein-rich medium that nurses feed to larvae [35], [36]. Furthermore, bees infected with *N. apis* have been shown to have a reduced lifespan, and were more likely to be found at the nest entrance engaged in guarding, or outside the nest performing orientation flights or foraging behavior [37]–[40].

In addition to changes in the rate of foraging onset, our data show that infection can disrupt the underlying physiology that regulates the age-specific expression of behaviors in workers. The trend in levels of Vg and JH showed an inverse relationship: when relative Vg transcript was at its lowest, JH titer was at its highest and vice versa for infected bees in cages. This finding is in agreement with research showing how Vg and JH act through positive feedback loops to negatively affect the synthesis of each other to regulate the age-specific expression of nursing or foraging behaviors [41]. In our cage studies, infected nurse-aged bees had an elevated JH titer that peaked at 8 days of age. This finding is corroborated by a recent study that showed higher levels of JH in bees infected with *N. ceranae* compared to bees infected with *N. apis* or uninfected controls [42]. We also saw an interaction between infection and age of bees on relative Vg transcript; however, the main effect of *N. ceranae* infection on Vg levels was not significant for bees in cages or field colonies. This finding is similar to a recent report that showed Vg transcript to be unchanged following infection with *N. ceranae* [21]. Our finding may suggest the strict inhibitory relationship that exists between Vg and JH may be an example of how a pathogen can decouple normally correlated behavioral syndromes by disrupting underlying physiology [43].

We saw considerable variability in the levels of relative Vg transcript and JH titer in our cage experiments. In fact, if cage of origin was modeled as a fixed effect in the ANOVA, a significant effect of cage could be observed. Modeling cages as a fixed effect is not statistically valid, but is mentioned here to illustrate a shortcoming of cage studies. The variability in bees among cages may be due to an absence of a queen, brood, or other environmental cues that workers typically encounter in the natural hive setting. It has been shown previously that contact pheromone produced by the mandibular glands of the queen suppresses JH biosynthesis in workers, resulting in low JH titer and a delay in foraging onset [44], [45]. Moreover, in the absence of primer pheromone produced by larvae, nurse-aged workers have lower Vg in fat body stores [46]. Interaction among workers may also affect behavioral development. In groups of bees lacking older individuals, 5−10% will display accelerated development [47]. If bees expressing a precocious phenotype, independent of treatment, were present in our cages they may have acted on other bees to suppress JH synthesis and behavioral maturation [48]. Placing bees in cages without the modulatory effects of queen and brood pheromones may not be the best way to study basic factors regulating behavioral development. Data from the field colonies, containing a queen and brood, better represent the effects of *N. ceranae* infection on Vg and JH.

The positive trend in relative Vg transcript seen in infected bees in cages over time may be due to variability among cages or it could be a host response induced by parasitism. Vitellogenin has life-extending properties, protecting workers from oxidative stress [49]. *Nosema ceranae*, like all microsporidia, lack mitochondria or have relict mitochondria-like structures with limited ATP-producing capability; therefore, it is characteristic of these fungi to aggregate near host mitochondria so as to exploit endogenous ATP [50]. Infection can result in the production of millions of spores in as little as 1−2 wks [51]. An infected bee may respond to this high rate of fungal proliferation by increasing levels of Vg to counter toxic reactive oxygen species produced from the elevated respiration rates in infected host midgut cells [52].

High annual mortality of honey bee colonies in the U.S. and worldwide due to persistent exposure to mite pests, poor nutrition, pesticides, and pathogens has created a need for alternative approaches to evaluate bee health. Diagnostic assays that establish the presence/absence of disease may provide limited information about the overall health of a colony [53], [54]. Currently, the standard method to diagnosis *Nosema* spp. infection is to estimate the average number of spores per bee by microscopy in a composite sample of 50–100 bees. Based on research presented here, it may be more informative to estimate the proportion of infected bees in a colony. If few bees are infected, the queen may be able to lay enough eggs to replace the loss, and uninfected bees may retain flexibility to revert to nurse bee physiology and behaviors as needed. On the other hand, if a high proportion of bees are infected, this flexibility in behavioral development may deteriorate, leading to colony decline. As it was not time-efficient to determine the proportion of infected bees, it may prove beneficial in future research to gauge colony health and productivity by examining genetic, endocrine, or other biochemical markers. Specifically, sampling for Vg or JH may provide a “medical record” of a colony’s ability to respond to infection by pathogens or other stressors. For example, poor nutrition could correlate with overall low levels of Vg in nurse-aged bees, as well as provide an indication of the antioxidant capacity of workers [14], [49].

In a honey bee colony, impairment of worker health through infection, as we have shown here, can result in alteration of behavioral performance and premature death. How the progression of an infectious disease impairs task performance and productivity of not only infected, but healthy workers, should be a goal of research exploring further the challenges to honey bee colony health.
